# Nintedanib Reduces Muscle Fibrosis and Improves Muscle Function of the Alpha-Sarcoglycan-Deficient Mice

**DOI:** 10.3390/biomedicines10102629

**Published:** 2022-10-19

**Authors:** Jorge Alonso-Pérez, Ana Carrasco-Rozas, Maria Borrell-Pages, Esther Fernández-Simón, Patricia Piñol-Jurado, Lina Badimon, Lutz Wollin, Cinta Lleixà, Eduard Gallardo, Montse Olivé, Jordi Díaz-Manera, Xavier Suárez-Calvet

**Affiliations:** 1 Neuromuscular Diseases Unit, Department of Neurology, Hospital de la Santa Creu i Sant Pau, Institut d’Investigació Biomèdica Sant Pau (IIB SANT PAU), 08041 Barcelona, Spain; 2 Departament of Medicine, Universitat Autònoma de Barcelona, 08041 Barcelona, Spain; 3 Cardiovascular Program ICCC, Hospital de la Santa Creu i Sant Pau Research Institute, IIB-Sant Pau, 08041 Barcelona, Spain; 4 Centro de Investigación Biomédica en Red en Enfermedades Cardiovasculares (CIBER-CV), Instituto de Salud Carlos III, 28222 Madrid, Spain; 5 The John Walton Muscular Dystrophy Research Centre, Newcastle University and Newcastle Hospitals NHS Foundation Trust, Newcastle upon Tyne NE1 3BZ, UK; 6 Boehringer Ingelheim, 88400 Biberach, Germany; 7 Centro de Investigación Biomédica en Red en Enfermedades Raras (CIBERER), Instituto de Salud Carlos III, 28222 Madrid, Spain

**Keywords:** sarcoglycanopathy, Sgca, muscular dystrophy, fibrosis, nintedanib, mice

## Abstract

Sarcoglycanopathies are a group of recessive limb-girdle muscular dystrophies, characterized by progressive muscle weakness. Sarcoglycan deficiency produces instability of the sarcolemma during muscle contraction, leading to continuous muscle fiber injury eventually producing fiber loss and replacement by fibro-adipose tissue. Therapeutic strategies aiming to reduce fibro-adipose expansion could be effective in muscular dystrophies. We report the positive effect of nintedanib in a murine model of alpha-sarcoglycanopathy. We treated 14 Sgca^-/-^ mice, six weeks old, with nintedanib 50 mg/kg every 12 h for 10 weeks and compared muscle function and histology with 14 Sgca^-/-^ mice treated with vehicle and six wild-type littermate mice. Muscle function was assessed using a treadmill and grip strength. A cardiac evaluation was performed by echocardiography and histological study. Structural analysis of the muscles, including a detailed study of the fibrotic and inflammatory processes, was performed using conventional staining and immunofluorescence. In addition, proteomics and transcriptomics studies were carried out. Nintedanib was well tolerated by the animals treated, although we observed weight loss. Sgca^-/-^ mice treated with nintedanib covered a longer distance on the treadmill, compared with non-treated Sgca^-/-^ mice, and showed higher strength in the grip test. Moreover, nintedanib improved the muscle architecture of treated mice, reducing the degenerative area and the fibrotic reaction that was associated with a reversion of the cytokine expression profile. Nintedanib improved muscle function and muscle architecture by reducing muscle fibrosis and degeneration and reverting the chronic inflammatory environment suggesting that it could be a useful therapy for patients with alpha-sarcoglycanopathy.

## 1. Introduction

Limb-girdle muscular dystrophies (LGMD) are a heterogeneous group of genetic diseases affecting skeletal muscle leading to progressive muscle weakness and irreversible disability [1]. Pathogenic variants in more than 30 genes have been described as the cause of LGMD, being the sarcoglycanopathies one of the most frequent forms, especially in the pediatric population [2,3,4,5,6,7]. There are four sarcoglycan genes: SGCA, SGCB, SGCD and SGCG, that encode for the alpha-, beta-, delta- and gamma-sarcoglycan protein, respectively, causing four recessive LGMD (LGMDR 3 to 6). Alpha-sarcoglycanopathy (LGMDR3), together with gamma-sarcoglycanopathy (LGMDR5), are the most frequent forms of sarcoglycanopathy, although depending on the population studied, the frequency of the type of sarcoglycanopathy may be different [4,7,8,9].

Sarcoglycans are transmembrane glycoproteins that form a tetrameric complex across the cell membrane of skeletal and cardiac muscle fibers [10,11,12]. This complex plays an important role in maintaining membrane integrity during the contraction and relaxation of skeletal muscle through its association with the dystroglycan complex, which links the subsarcolemmal protein dystrophin to the basement membrane [12,13]. Pathogenic variants in any of the four sarcoglycan genes disrupt the whole complex, leading to a loss of muscle membrane integrity and its rupture after each muscle contraction [13,14].

The process of muscle fiber degeneration in muscular dystrophies has been well characterized [15]. It has been reported and is well-accepted that muscle membrane instability leads to continuous muscle fiber damage, leading to several cycles of myofiber degeneration and regeneration. This process produces a modification of the muscle microenvironment both at a cellular and molecular level. On the one hand, there is a continuous activation of satellite cells, that aim to regenerate the injured muscle fibers, but on the other hand, there is a persistent infiltration of inflammatory profibrotic cells, mainly M2 macrophages, which release several cytokines orchestrating the degenerative process [16,17,18]. It has been suggested that these cytokines, mainly TGF-b and CTGF, among many others, activate the proliferation and differentiation of fibroadipogenic progenitor cells (FAPs), which are key in the expansion of the fibrotic and adipose tissue replacing muscle fibers [19]. The expansion of fibrotic and adipose tissue has negative consequences for the muscle, including their lack of contractile properties leading to muscle weakness and contractures and the impaired satellite cells’ ability to efficiently regenerate the damaged muscles. Eventually, the majority of muscle fibers are lost, and the skeletal muscles are replaced by fibrotic and adipose tissue [18,20].

Among the therapeutic strategies being developed for patients with muscular dystrophies, drugs interfering with the expansion of fibro-adipose tissue have been already proposed. For example, drugs decreasing TGF-β activity have been shown to reduce the amount of fibrous tissue but also to increase inflammatory infiltrates in the muscles of murine models of muscular dystrophies [21,22]. These initial results promoted research to explore the role of other growth factors on muscle fibrosis and treatments that counteract [23,24,25,26,27]. Alternatively, interfering with the chronic inflammatory process that enhances the expansion of fibrotic tissue has also been proposed as a therapeutic option [26,28,29,30]. In this sense, treatment with corticosteroids slows down disease progression and improves survival in patients with Duchenne muscular dystrophy (DMD) and is considered part of the standard of care for these patients [31]. More recently, drugs blocking the P2X7 purinoreceptor, which is over-expressed in dystrophic muscle and plays a role in the induction of immune response, have been shown to reduce the inflammatory response and consequently muscle fibrosis in mdx and Sgca murine models [32].

Within the different growth factors that could be involved in the fibrotic process in muscular dystrophies, the family of platelet-derived growth factors (PDGF) has centered our interest in the last few years. We demonstrated that PDGF-AA is increased in muscle samples of patients with DMD and PDGF-AA enhances fibroblast and FAP cell proliferation, migration and collagen expression [23,24]. The PDGF receptor has been targeted with different treatments, including imatinib and crenolanib which are tyrosine kinase inhibitors (TKi), with a positive effect in reducing fibrosis in the muscles, but leading to severe adverse effects [33,34]. Nintedanib is a second-generation TKi targeting PDGF α and β receptors (PDGFRA and PDGFRB), fibroblast growth factor receptor (FGFR) 2 and 3 and vascular endothelial growth factor receptor (VEGFR) 1–3 [35]. The anti-fibrotic activity of nintedanib has been demonstrated in vitro in primary lung fibroblasts from patients with idiopathic pulmonary fibrosis (IPF) but also in muscle fibroblasts from DMD and in dermal fibroblasts from patients with systemic sclerosis, and in vivo in animal models of several fibrotic diseases [24,36,37]. Moreover, nintedanib is approved for the treatment of IPF, which is characterized by fibrosis of the lungs [38,39,40].

In this study, we evaluated the effect of nintedanib on muscle fibrosis and modulation of the inflammatory response as well as on motor function in a mouse model of alpha-sarcoglycanopathy.

## 2. Materials and Methods

### 2.1. Mouse Model

**Figure 1:**
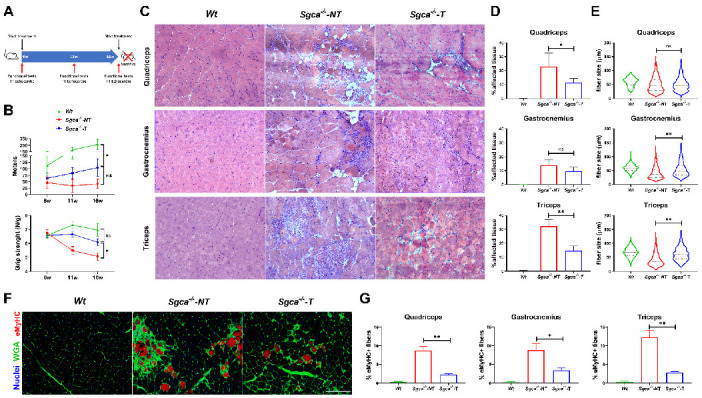
**Effect of nintedanib on muscle function and architecture.** (**A**) Scheme of the experimental design and the treatment of mice with nintedanib. (**B**) Results of the muscle functional tests at the different time points. Top panel, treadmill test showing a significant improvement of the covered distance of Sgca^-/-^-T compared to Sgca^-/-^-NT. Bottom panel, grip strength test showing that muscle strength significantly improved after treatment with nintedanib. (**C**) Representative pictures of the hematoxylin and eosin (H-E) staining of muscle sections from quadriceps, gastrocnemius and triceps of all *Wt*, Sgca^-/-^-NT and Sgca^-/-^-T mice. (**D**) Histological assessment of the affected muscle tissue including muscle necrosis, fibrosis and inflammation in *Wt*, Sgca^-/-^-NT and Sgca^-/-^-T mice. (**E**) Cross-sectional muscle fiber area quantification in muscle sections from quadriceps, gastrocnemius and triceps. (**F**,**G**) Effect of nintedanib on the number of regenerating muscle fibers. Representative images of eMyHC staining are shown (**F**) and the percentage eMyHC positive fibers over total fibers are shown (**G**). TT: Transthoracic. Genetic background mouse strain C57BL (*Wt*); *n* = 6, no-treated Sgca^-/-^ mice (Sgca^-/-^-NT), *n* = 14; nintedanib-treated Sgca^-/-^ mice (Sgca^-/-^-T). Data are expressed as means ± SD. w = weeks; * *p* < 0.05 and ** *p* < 0.001. Scale bar = 200 μm. ns indicates no significant difference.

Six-week-old B6.129S6-Sgcatm2Kcam/J mice (JAX stock #008275) [41] (*n* = 14, 7 male and 7 female) were treated with 50 mg/kg of Nintedanib (from now on, Sgca^-/-^-T) (Boehringer Ingelheim, Ingelheim, Germany) or vehicle (from now on, Sgca^-/-^-NT; *n* = 14, 8 male and 6 female). The concentration of Nintedanib (50 mg/kg twice daily) was chosen based on previous in vivo studies, in which this dose was chosen for its optimal efficacy in mice [37,42]. Mice were treated every 12 h for 10 weeks. Six C57BL6 healthy mice were also included as controls (from now on Wt; *n* = 6, 4 male and 2 female). Nintedanib was solubilized in sterile ultra-pure water (Braun, Rubi, Spain) and administered by gavage. Functional motor and echocardiographic studies were performed in all animals of the three groups before starting treatment (baseline), in the middle of the treatment period (week 5) and at the end of treatment (week 10). At 16 weeks of age animals were euthanized and the quadriceps, gastrocnemius, triceps and heart muscles were collected and processed for analysis (Figure 1A). All animal procedures were performed according to the National Institute of Health Guidelines for the Care and Use of Laboratory Animals [43] and were approved by the Hospital de la Santa Creu i Sant Pau Animal Ethics Committee. To control the safety of the drug, weight control was performed by monitoring every two days. Progressive weight loss or loss of more than 2 g of weight in successive controls was established as a criterion for discontinuation of treatment.

### 2.2. In Vivo Muscle Function

The effect of the treatment with nintedanib on the maximal running capability of mice was assessed using a treadmill (Columbus Instruments Exer 3/6 Treadmill, Columbus, OH, USA) following the standard guidelines proposed by TREAT-NMD. In summary, after the acclimatation period, mice were placed on the treadmill at a starting running speed of 5 m/min accelerating 1.5 m/min until reaching a final velocity of 36 m/min. The test was finished when mice became exhausted, defined as the inability of mice to run for 10 s despite gentle push with the hand in the running direction, or if the mice touched the end of the belt more than 40 times. This protocol was adapted from TREAT-NMD guidelines (https://treat-nmd.org/resources-support/research-overview/preclinical-research/experimental-protocols-for-dmd-animal-models/; accessed on 9 September 2022) and from previously published works [44,45]. Maximum grip strength in the forelimbs was assessed using a grip strength meter (Columbus Instruments Grip Strength Meter, Columbus, OH, USA). Five measurements were recorded per animal and time point. The three highest values were selected and normalized for body weight (Newtons/grams).

### 2.3. Echocardiography

Transthoracic echocardiography was performed using the Vevo 2100 ultrasound system (Visualsonics, Toronto, Canada) equipped with a high-frequency (30 MHz) linear array transducer. Animals were placed supine on an electrical heating pad at 37 °C under light isoflurane anesthesia (2% isoflurane). Continual ECG monitoring was obtained via limb electrodes. Two-dimensional and M-mode images were obtained in parasternal long-axis and short-axis views, respectively. Careful attention was paid to image depth, width and gain settings, in order to optimize image quality. All views were digitally stored in cine loops consisting of 300 frames. Measurements of LV interventricular septal thickness (IVS), LV internal dimensions (LVID) and the thickness of the LV posterior wall (LVPW) and the LV anterior wall (LVAW) at diastole and systole (IVSd, LVIDd, LVPWd, LVAWd and IVSs, LVIDs, LVPWs, LVAWs, respectively) were obtained. Any images obtained with suboptimal physiological parameters were excluded from the analysis. Subsequent analysis was performed by an experienced cardiologist blind to genotype and treatment. Ejection fraction (EF), fractional shortening (FS), stoke volume (μL) and cardiac output were determined.

### 2.4. Histology and Immunofluorescence

Frozen muscle sections of 7 µm were obtained using a Leica cryostat (Leica Microsystems, Wetzlar, Germany). For the evaluation of the compromise along each muscle, the following procedure was performed. Starting from the central segment of each muscle, 60–80 slices were obtained that were distributed both for histology, immunofluorescence and Fast Green–Sirius Red studies, so that a representation of various levels of each muscle was obtained with the aim of improving the interpretation of global involvement of each muscle.

For histological evaluation sections of the quadriceps, gastrocnemius, triceps and heart were stained with hematoxylin and eosin (H-E) following standard protocol. The analysis of the H-E staining was performed by assessing the percentage of tissue affected by necrosis, fibrosis and inflammation evaluating the entire muscle section, as previously described [46,47]. For immunofluorescence studies, tissue sections were fixed with acetone for 5 min, washed with PBS and incubated with blocking solution (Cat. Nº SC516214) (UltraCruz Blocking Reagent—Santa Cruz Biotech) for 30 min. Tissue sections were incubated with goat polyclonal anti-Collagen I-UNLB (Cat. Nº 1441-01) (Southern Biotech, Birmingham, AL, USA), goat polyclonal anti-PDGFRα (Cat. Nº AF1062-SP) (R&D Systems, Minneapolis, MN, USA), rat monoclonal anti-F4/80 BM8 (Cat. Nº 14-4801-82) (ThermoFisher, Waltham, MA, USA). The Mouse on Mouse (M.O.M.^®^) Immunodetection Kit (BMK2202) (Vector Laboratories, Newark, CA, USA) was used for the immunofluorescence studies using mouse monoclonal anti-embryonic Myosin heavy chain (eMyHC) (Cat. Nº F1.652) (DSHB, Houston, TX, USA). Appropriate Alexa-conjugated secondary antibodies were used at 1/400 (ThermoFisher, Waltham, MA, USA). Images were obtained with an Olympus BX51 microscope coupled to a DP72 camera (Olympus, Tokyo, Japan). ImageJ software was used to quantify the positive area according to negative controls. A minimum of six independent fields per staining were quantified [48].

### 2.5. Fast Green-Sirius Red

Collagen content in quadriceps, gastrocnemius, triceps muscles and the heart was quantified by the Fast Green-Sirius Red (FG-SR) technique, as previously described [49,50]. Briefly, ten cryosections were collected in a microtube and sequentially incubated with a solution containing 0.1% Fast green in saturated picric acid (Cat. Nº F7252-5G) (Sigma-Aldrich, St. Louis, MO, USA) for 30 min, washed with distilled water and incubated with 0.1% Fast green and 0.1% Sirius red (Cat. Nº O0625) (Sigma, St. Louis, MO, USA) in saturated picric acid for 90 min. The sections were washed with distilled water and gently shaken in a solution of 0.1 M NaOH (Cat. Nº 106462) (Merck, Burlington, MA, USA) in absolute methanol (1:1) (Cat. Nº 67-56-1) (Sigma-Aldrich) for 20 min. Absorbance was measured in a spectrophotometer at 540 and 605 nm wavelengths. Total protein and collagen equivalences of the obtained absorbance values were calculated afterwards. Collagen values were expressed as the percentage of the total protein in the muscle sample.

### 2.6. Cytokine and Chemokine Arrays

We assessed the effect of nintedanib on the concentration of several cytokines and chemokines involved in the muscle inflammatory process by using the Proteome Profiler Mouse Cytokine Antibody Array Kit (Cat. Nº ARY006) (R&D Systems) which, simultaneously, detects 40 cytokines, chemokines and acute phase proteins (Table S1). We assessed the concentration of these molecules in muscle samples of four Sgca^-/-^-T mice, four Sgca^-/-^-NT mice and three Wt mice that were randomly selected. A fragment of the quadriceps muscle was disrupted using TissueRuptor II (QIAGEN, Germantown, MD, USA), incubated with RIPA lysis and a protein extraction buffer (Cat. Nº R0278-50ML) (Sigma, St. Louis, MO, USA), containing protease inhibitor cocktail (Cat. Nº P8340-1ML) (Sigma, St. Louis, MO, USA) and quantified using Protein Assay Reagent B (Cat. Nº 500-0114) (Bio-Rad, Hercules, CA, USA), according to manufacturer’s recommendations. A total of 100 ug of protein lysate per animal was assayed. Data were expressed as a fold change compared to the Wt group. The protein network for bioinformatics analysis of the modulated cytokines was generated using the STRING^®^ version 11.5 platform (ELIXIR, Hinxton, Cambridgeshire, UK) [51]. Data generated by the STRING analysis, including the Reactome^®^ (version 82) pathways (ELIXIR, Hinxton, Cambridgeshire, UK), were adjusted by using the false discovery rate (FDR).

### 2.7. Gene Expression Profiling

The effect of nintedanib on the expression of fibrosis-related genes was assessed by real-time quantitative PCR (RT-qPCR) using the TaqMan^®^ Array Mouse Fibrosis (Cat. Nº 4413255) (Applied Biosystems, Foster City, CA, USA) which allows the analysis of 84 fibrosis-related genes and 12 endogenous gene candidates (Table S2). We isolated a total RNA from the same mouse muscle samples, randomly selected for the array studies. Briefly, RNA was extracted from quadriceps muscle from each included animal using TRIzol™ Reagent (Cat. Nº 15596018) (Invitrogen™, Carlsbad, CA, USA) in conjunction with the PureLink™ RNA Mini Kit (Cat. Nº 12183018A) (Invitrogen™, Carlsbad, CA, USA). Contaminating DNA was digested with PureLink^®^ DNase (Cat Nº 12185010) (Invitrogen™, Carlsbad, CA, USA). RNA was quantified using a nanodrop ND-1000 spectrophotometer (Nanodrop Technologies Inc., Wilmington, DE, USA). In all samples, 6 μg of total RNA was reverse-transcribed to complementary DNA (cDNA) using the High-Capacity cDNA Reverse Transcription Kit (Cat. Nº 4368814) (Applied Biosystems, Foster City, CA, USA). RT-qPCR was performed using the Fast TaqMan^®^ Universal PCR Master Mix (Cat. Nº 4352042) (Applied Biosystems) and a 7500 Fast Real-Time PCR System (Applied Biosystems). Relative quantification was performed using the comparative Ct method with the Sequence Detection System (SDS) software (Applied Biosystems) [52]. A *Tbp* gene was used as an endogenous control as it showed stable expression across the different experimental groups (Figure S1).

### 2.8. Statistical Analysis

Quantitative variables were analyzed using the Shapiro–Wilk test to verify their normal distribution. Comparison between the different subgroups of mice was performed using a one-way ANOVA test. When ANOVA revealed significant differences, the Tukey post hoc test was performed. The statistical analyses were calculated using GraphPad Prism 8 software (GraphPad Software, San Diego, CA, USA). The significance level was set at *p* < 0.05.

## 3. Results

### 3.1. Nintedanib Improves Muscle Endurance and Strength in Sgca^-/-^ Mice

We evaluated the effect of nintedanib on muscle function using two different tests. The treadmill test is considered a muscle endurance test that evaluates the maximum running capability. At baseline, when the mice were 6 weeks old and before starting the treatment, no statistically significant differences were observed in the distance covered by Sgca^-/-^-T, Sgca^-/-^-NT and Wt mice. At 11 weeks of age, Wt mice showed a greater increase in the distance covered, compared with the Sgca^-/-^-NT or the Sgca^-/-^-T mice. However, at that time point, there were already statistically significant differences in the running distance between Sgca^-/-^-T and Sgca^-/-^-NT mice (83.3 vs. 34.1 m, two-way ANOVA test–Tukey test, *p* < 0.001). At 16 weeks, after 10 weeks of treatment, the differences between Sgca^-/-^-T and Sgca^-/-^-NT were bigger (108.9 vs. 41.9 m, two-way ANOVA test–Tukey test, *p* < 0.001) (Figure 1B).

To assess muscle strength, we evaluated the maximum grip strength in the forelimbs. At baseline, there were no differences amongst groups. At 11 weeks, after 5 weeks of treatment, we observed a 1.2-fold reduction in muscle strength in Sgca^-/-^-NT mice, compared to Sgca^-/-^-T mice (two-way ANOVA test–Tukey test, *p* = 0.005). At that time point, there were no significant differences in grip strength between the Wt and Sgca^-/-^-T groups (two-way ANOVA test–Tukey test, *p* = 0.165). Similarly, at 16 weeks Sgca^-/-^-NT mice continued to show a 1.2-fold significant reduction in muscle strength compared to Sgca^-/-^-T mice (two-way ANOVA test–Tukey test, *p* = 0.016). Finally, non-significant differences were observed between Sgca^-/-^-T mice and Wt mice, although muscle strength was lower in Sgca^-/-^-T mice (two-way ANOVA test–Tukey test, *p* = 0.25). (Figure 1B).

No significant difference was observed between Sgca^-/-^-NT, Sgca^-/-^-T and Wt mice in weight gain after 10 weeks of treatment (two-way ANOVA test–Tukey test, *p* = 0.10), however, a greater weight gain was observed in Sgca^-/-^-NT compared to Sgca^-/-^-T mice (29.9% vs. 23.3%) (Figure S2).

### 3.2. Effects of Nintedanib on Cardiac Function and Ventricular Remodeling in Wt and Sgca^-/-^ Mice

Diastolic and systolic functions were assessed by echocardiography at baseline, five and 10 weeks of treatment. At 10 weeks of treatment, when the animals were 16 weeks old, we observed differences in the systolic function of Sgca^-/-^-NT mice compared to Wt mice. However, at that time point, Sgca^-/-^-T mice showed similar systolic functions as untreated Sgca^-/-^-NT mice. Left ventricle (LV) wall thickening assesses global LV function in the absence of abnormal wall motion. Interestingly, the LV anterior wall of Sgca^-/-^-NT mice was thicker than Wt littermates, and this morphologic trait was mitigated in Sgca^-/-^-T mice, suggesting a potential role of nintedanib in the cardiac ventricle wall size (Figure S3A). However, functional analysis of the heart by the analyses of the ejection (EF%) and shortening fraction (FS%) showed that both Sgca^-/-^-T and Sgca^-/-^-NT mice have worse performances than their Wt littermates, suggesting that Sgca^-/-^ null mice develop a cardiac dysfunction early in their life. Despite these alterations at the functional level, we did not find structural differences in heart sections stained with H-E between Wt and Sgca^-/-^-NT nor an increase in collagen in the hearts of Wt, Sgca^-/-^-T or Sgca^-/-^-NT mice (Figure S3B). * *p* < 0.05, ns = no significant difference.

### 3.3. Nintedanib Improves Skeletal Muscle Architecture

We assessed the effect of 10 weeks of nintedanib treatment on muscle architecture by quantifying the amount of tissue affected, including areas of necrosis, fibrosis and inflammation by H-E staining, as previously described [46,47] (Figure 1C). We found a significant reduction in the percentage of affected tissue between the Sgca^-/-^-NT and Sgca^-/-^-T mice in the quadriceps (ANOVA test–Tukey test, *p* = 0.02) and triceps (ANOVA test–Tukey test, *p* < 0.001) muscles. A tendency towards a reduced affected tissue was also observed in the gastrocnemius (ANOVA test–Tukey test, *p* = 0.09) (Figure 1D). Significant differences in fiber size between the Sgca^-/-^-NT and Sgca^-/-^-T mice were found in the gastrocnemius (Mann–Whitney test, *p* < 0.001) and triceps (Mann–Whitney test, *p* < 0.001) (Figure 1E). In detail, muscle fiber size was more homogeneous, and fibers tended to be smaller. To investigate the effect of nintedanib in muscle regeneration, we quantified the number of fibers expressing eMyHC, a marker of regenerative muscle fiber, per every 100 muscle fibers, and observed a significant decrease in the percentage of eMyHC + fibers in all muscles assessed in Sgca^-/-^-T, compared with Sgca^-/-^-NT mice (ANOVA test—Tukey test, *p* < 0.001) (Figure 1F,G).

### 3.4. Nintedanib Reduces Muscle Fibrosis in Sgca^-/-^ Mice

**Figure 2:**
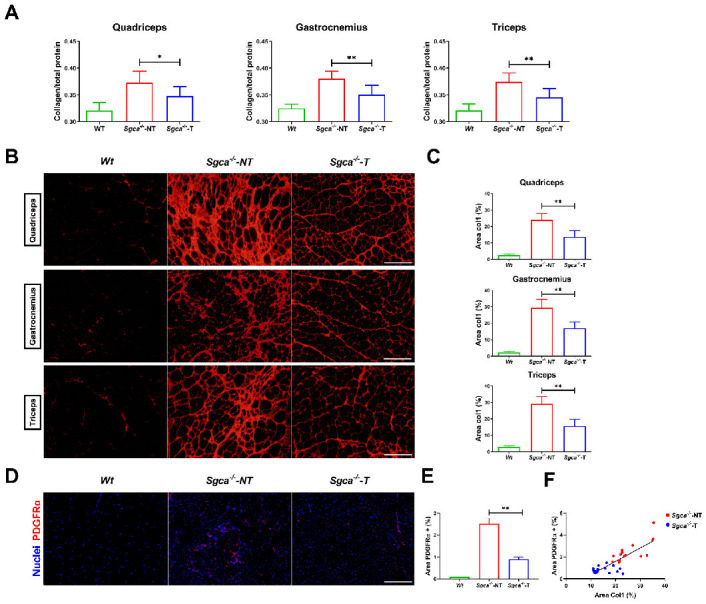
**Nintedanib reduces muscle fibrosis in Sgca^-/-^ mice.** (**A**) Nintedanib-treated mice showed a significant reduction in the total collagen analysis by Fast Green technique in quadriceps, gastrocnemius and triceps. (**B**,**C**) Analysis of the collagen I area by immunofluorescence of muscle sections demonstrated a significant reduction in quadriceps, gastrocnemius and triceps after treatment with nintedanib. Genetic background mouse strain C57BL (*Wt*); *n* = 6, non-treated Sgca^-/-^ mice (Sgca^-/-^-NT), *n* = 14; nintedanib-treated Sgca^-/-^ mice (Sgca^-/-^-T). (**D**,**E**) Analysis of the amount of FAPs cells in *Wt*, Sgca^-/-^-NT and Sgca^-/-^-T mice by PDGFRα immunofluorescence. A representative image is shown. (**F**) Correlation analysis between PDGFRα area and Collagen I in all muscles analyzed (quadriceps, gastrocnemius and triceps). Results of (**D**,**E**) are from randomized samples of Sgca^-/-^ mice (Sgca^-/-^-NT), *n* = 5; and nintedanib-treated Sgca^-/-^ mice (Sgca^-/-^-T), *n* = 5. Col1: Collagen I. Data are expressed as means ± SD. * *p*< 0.05 and ** *p*< 0.001. Scale bar = 200 μm.

We quantified the total collagen content in the muscle in relation to the total protein content by the Fast Green-Sirius Red technique [49,50]. We observed a significant reduction in the collagen/total protein ratio in quadriceps (ANOVA test–Tukey test, *p* = 0.002), gastrocnemius (ANOVA test—Tukey test, *p* < 0.001) and triceps (ANOVA test–Tukey test, *p* < 0.001) of Sgca^-/-^-T mice versus Sgca^-/-^-NT mice (Figure 2A).

Then, we analyzed the area occupied by Collagen-I using IF in muscle sections (Figure 2B). We observed an increase in the Collagen-I immunolabelled area of 21.3% in the quadriceps, 26.5% in the gastrocnemius and 25.6% in the triceps in Sgca^-/-^-NT mice compared to aged-matched Wt mice. Nintedanib significantly reduced the Collagen-I area in the quadriceps (−10.2%), gastrocnemius (−12.5%) and triceps (−13.0%) of Sgca^-/-^-T mice compared with Sgca^-/-^-NT mice (Figure 2C).

We assessed the effect of nintedanib on the PDGFRα area using IF which is a well-known marker of FAPs. As expected, we observed an increase in the percentage of the PDGFRα positive area in the Sgca^-/-^-NT mice compared to Wt (2.5% vs. 0.1) (Figure 2D,E). Nintedanib significantly reduced the PDGFRα positive area in Sgca^-/-^-T compared to Sgca^-/-^-NT mice in quadriceps (ANOVA test—Tukey test, *p* < 0.001), gastrocnemius (ANOVA test–Tukey test, *p* = 0.001) and triceps (ANOVA test–Tukey test, *p* = 0.02). Moreover, we observed a significant correlation between the fibrotic Collagen-I and PDGFRα positive area (Pearson correlation, *p* < 0.001, R^2^ = 0.67) (Figure 2F).

**Figure 3:**
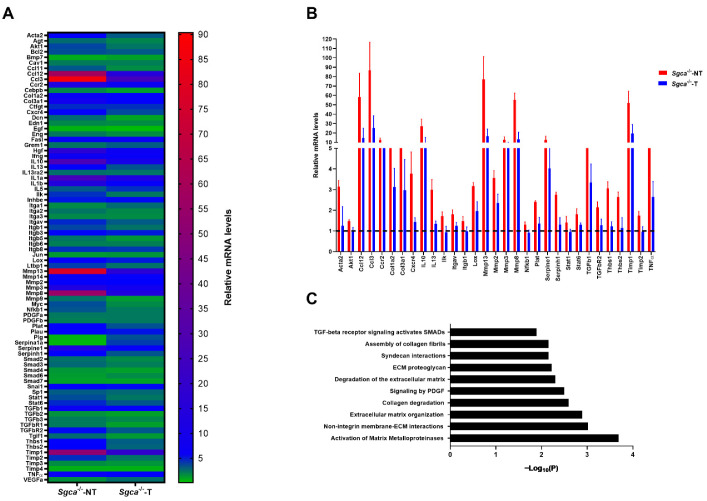
**Nintedanib reduces gene expression of fibrosis-related genes in muscle.** (**A**) Heatmap of relative mRNA expression of 84 analyzed genes. The color gradient represents the relative ΔΔCt value. (**B**) Schematic representation of the overexpressed genes in the Sgca^-/-^ mice that are significantly reduced after treatment with nintedanib. (**C**) Schematic representation of Reactome^®^ pathways that are significantly reduced in nintedanib-treated mice. Genetic background mouse strain C57BL (*Wt*); *n* = 3, no-treated Sgca^-/-^ mice (Sgca^-/-^-NT), *n* = 4; nintedanib-treated Sgca^-/-^ mice (Sgca^-/-^-T), *n* = 4. Data are expressed as means ± SD and *Tbp* gene was used as endogenous control.

To examine further the role of nintedanib on muscle fibrosis in Sgca^-/-^ mice, we performed experiments at the protein and transcriptional level. We confirmed the antifibrotic effect of nintedanib at the transcriptional level by assessing expression levels of a large panel of extracellular matrix and fibrosis-related genes. We observed a generalized reduction in the expression of the genes involved in fibrosis between Sgca^-/-^-T and Sgca^-/-^-NT mice (Figure 3A). In detail, a significant upregulation (>1.3 fold-change) in 46 out of 84 genes analyzed was found in Sgca^-/-^-NT mice compared to Wt mice, which was significantly reduced in 31 genes after treatment with nintedanib. The genes that showed a decreased expression after nintedanib treatments were *Ccl3, Mmp13, Ccl12, Mmp8, Timp1, IL10, Mmp3, Ccr2, Serpine1, Col3a1, Tgfb1, Tnf, Col1a2, Cxcr4, Mmp2, Lox, Acta2, Thbs1, Il13, Serpinh1, Thbs2, Plat, Tgfbr2, Stat6, Itgav, Timp2, Ilk, Akt1, Itgb1, Stat1* and *Nfkb1* (Figure 3B). To better understand the fibrotic molecular pathways modulated by nintedanib, we analyzed our results using Reactome^®^ software and found that matrix metalloproteinases, molecules involved in the non-integrin membrane and an extracellular matrix interactions, molecules involved in extracellular matrix organization and collagen degradation and PDGF signaling-related molecules were the main cellular processes modulated by nintedanib treatment (Figure 3C).

### 3.5. Nintedanib Modulates Chronic Muscle Inflammation in the Sgca^-/-^ Mice

**Figure 4:**
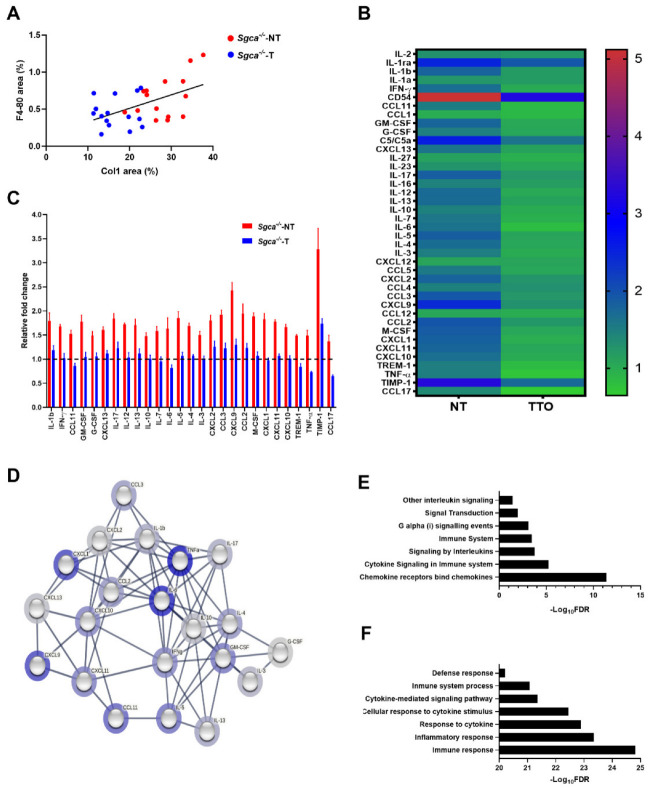
**Effect of nintedanib on muscle inflammation in Sgca^-/-^ mice.** (**A**) A positive correlation was observed between the quantity of infiltrating macrophages (F4-80 positive area) and collagen I (Col1 positive area) in all muscle analyzed (quadriceps, gastrocnemius and triceps) from randomized samples of Sgca^-/-^ mice (Sgca^-/-^-NT), *n* = 5; and nintedanib-treated Sgca^-/-^ mice (Sgca^-/-^-T), *n* = 5. (**B**) Heatmap representation of the expression of 40 inflammatory mediators in the muscle of all groups of animals relative to the Wt mice. (**C**) Significantly reduced inflammatory molecules after treatment with nintedanib. Red bar corresponds to untreated mice. Blue bar corresponds to nintedanib-treated mice. (**D**) Functional annotation of inflammatory molecules analyzed. Lines in the network represent the cytokine interactions. Halo color is based on fold change of protein expression in Sgca^-/-^ mice relative to Sgca^-/-^ nintedanib-treated mice. (**E**,**F**) Schematic representation of Reactome^®^ pathways (**E**) and biological processes related to cytokine modulation (**F**) that are significantly reduced in nintedanib-treated mice. Genetic background mouse strain C57BL (*Wt*); *n* = 3, no-treated Sgca^-/-^ mice (Sgca^-/-^-NT), *n* = 4; nintedanib-treated Sgca^-/-^ mice (Sgca^-/-^-T), *n* = 4. Data are expressed as means ± SD.

The presence of inflammatory infiltrates is a hallmark of the muscle degeneration processes in muscular dystrophies. To evaluate the role of nintedanib in this process we first assessed the macrophagic infiltration in muscles by F4/80 antibody staining, a well-known marker of murine macrophages. We observed a reduction in the F4/80 staining between the Sgca^-/-^-T and Sgca^-/-^-NT mice in all muscles, although these differences significantly only reached the triceps (ANOVA test–Tukey test, *p* = 0.02). A mild correlation between the percentage of Collagen-I immunolabeling and F4/80 positivity was observed (Pearson correlation, *p* = 0.003, R^2^ = 0.27) (Figure 4A). In addition, we assessed the expression of 40 cytokines and chemokines involved in the inflammatory process in muscle samples and observed a generalized reduction in the expression of several cytokines and chemokines (Figure 4B). In detail, in the Sgca^-/-^-NT mice 32 out of 40 cytokines and chemokines analyzed were significantly upregulated (>1.3-fold change) compared to Wt mice. Nintedanib treatment resulted in a significant reduction in 27 out of these 32 upregulated cytokines including TIMP-1, CXCL9, CCL2, CCL3, M-CSF, IL-5, IL-17, CXCL1, CXCL2, IL-1b, GM-GSF, CXCL11, IL-12, IL-13, IL-4, IFN-γ, CXCL10, IL-6, CXCL13, IL-7, CCL11, IL-3, TREM-1, TNF-α, G-CSF, IL-10 and CCL17 (Figure 4C). The functional and physical relationship of these cytokines based on fusion and neighborhood evidence, and the existing databases after the bioinformatics analysis (STRING^®^) indicated that most of the downregulated cytokines are involved in chemokine receptors bind chemokines, cytokine signaling in immune system pathway, signaling by interleukins, Immune System, G alpha (i) signaling events, signal transduction and other interleukin signaling pathways (Figure 4D,E). These pathways are involved in the regulation of the biological process of immune response, general inflammatory response, cellular response to cytokine stimulus, cytokine-mediated signaling pathway, immune system process and defense response (Figure 4F).

## 4. Discussion

We report the positive therapeutic effect of nintedanib in a murine model of alpha-sarcoglycan-deficient muscular dystrophy. Our results demonstrate that nintedanib reduces muscle fibrosis and modifies the existing proinflammatory muscle microenvironment, leading to improved muscle endurance and strength and attenuating the dystrophic phenotype of the mice. These data are especially relevant at present, as gene delivery-based therapies are under development and interventional clinical trials in patients with alpha-sarcoglycanopathies are being planned [53,54,55]. In this scenario, drugs reverting the profibrotic muscle microenvironment could be prescribed alone or in combination with gene therapies to preserve the muscle structure in patients with muscular dystrophies.

In muscular dystrophies, there are continuous episodes of muscle degeneration and regeneration leading to persistent infiltration of inflammatory cells [17,18]. It has been hypothesized that inflammatory cells release multiple cytokines and growth factors that contribute to the fibrotic process by activating FAPs cells and fibroblasts resident in the skeletal muscle [17,19,34,56]. This mechanism is common to many muscular dystrophies but also to other myopathies, especially when the muscle fiber membrane is fragile, as is the case of sarcoglycanopathies or DMD. If successful, the new gene delivery strategies under development will provide a healthy copy of the missing gene to several muscle fibers, targeting the primary molecular defect. However, the increase in fibrotic and tissue replacement and loss of muscle fibers hinders the efficacy of gene therapies prompting the research in combined treatments with pro-regenerative and/or antifibrotic drugs that could maintain the muscle architecture. Nintedanib modulates FGF, PDGF and VEGF receptors involved in the complex molecular network orchestrating the fibrotic process in muscular dystrophies. It also inhibits fibroblast proliferation and migration in vitro and reduces fibrosis in animal models of pulmonary fibrosis or systemic sclerosis [36,37]. We have already shown that nintedanib reduces the proliferation and migration of fibroblasts obtained from DMD patients’ muscle samples and demonstrated an antifibrotic effect in 10-month-old mdx mice [24]. However, major functional changes were not found, prompting us to test nintedanib in a more severe murine model that better reflects the human disease pathology. We are now showing that nintedanib reduces fibrosis and modulates inflammation in the Sgca^-/-^ mice, improving the muscle strength. These results suggest that nintedanib could be considered as a potential effective antifibrotic therapy for patients with muscular dystrophies and tested in clinical trials.

Sgca^-/-^ mice show a more severe muscle phenotype than mdx mice. Indeed, natural history studies in Sgca^-/-^ mice show that the muscle function is already impaired at 8 weeks and a severe muscle impairment is detectable at 14 to 16 weeks [46,47]. These results were confirmed in our study. We observed early impairment of muscle function tests at an age of 11 weeks, which progressed in the non-treated mice until 16 weeks of age. Nintedanib treatment slowed down the progressive muscle impairment and the treated mice displayed a better functional outcome than age-matched not treated animals. However, nintedanib treatment did not abrogate muscle impairment completely to the control level. This improvement was associated with a better-preserved muscle architecture consisting of a lower number of necrotic fibers and a reduced number of regenerative fibers, less fibrosis and inflammation.

In recent years, FAPs cells are considered to be key in muscle fibrosis and adipogenesis, but they could also have a role in orchestrating the whole degenerative process by regulating satellite cell or macrophage’s function. In this sense, the number of FAPs increases in muscle samples of patients with muscular dystrophies and correlates with the fibrotic area of the muscles affected [57]. We have observed a reduction in the number of cells expressing PDGFRα, which is a well-accepted canonical marker of FAPs cells [34,58], associated with an 11.9% reduction in the collagen-stained area in Sgca^-/-^-treated mice compared to untreated mice.

In addition to the histologic studies, we assessed the expression levels of 84 genes involved in the fibrotic process. Our results suggest that nintedanib has an impact on several pathways involved in fibrosis, not only the PDGF pathway. For instance, Col1a2 and Col3a1, the two main components of the extracellular matrix, and TGFB1 and TGFBR2, central components of the TGF-b pathway [21,59], are decreased in treated animals’ muscle samples. Several metalloproteinases, but also the tissue inhibitors of metalloproteinases 1 and 2 (TIMP1 and TIMP2), which participate in the remodeling process of the extracellular matrix and have been shown to be upregulated in muscles and plasma from patients with DMD [60], were also decreased after nintedanib treatment. This data suggests that the inhibition of the PDGFR-a by nintedanib reduced the increase in muscle fibrotic expansion. Indeed, we have recently observed that PDGF-AA increases proliferation, migration and collagen expression by FAPs [23]. Reducing the activation of FAPs could result in a reduced fibrotic reaction and a more conserved muscle architecture with less necrotic fibers and regenerative fibers [57,61]. In addition, in this study, we observed a reduction in the expression of some genes that are also involved in inflammation. For example, a massive reduction in the expression of CCL3 and IL-13 was observed. CCL3 has been considered a macrophage chemoattractant [62] and IL-13 has been associated with the change of macrophage phenotype towards macrophage M2 profibrotic [20]. This could suggest that nintedanib, through its main antifibrotic role, can also modulate chronic inflammation secondary to chronic muscle damage that occurs in muscular dystrophies.

We assessed the expression levels of 40 cytokines and chemokines involved in the inflammatory process observing a reduction in the expression of several of these. For example, we observed a substantial reduction in the expression of CCL2, CCL3, CXCL10 and CXCL11 which are involved in the chemotaxis of immune cells [62]. Moreover, we observed a reduction in IL-4 and IL-13 cytokines associated with the switch of the macrophage phenotype towards an M2 profibrotic status [20]. This reinforces the hypothesis that a reduction in the fibrotic process could lead to related inflammatory activity resulting in more preserved muscle structure. The levels of molecules involved with satellite-cell proliferation and differentiation, such as TNFα or IL-6, were also modulated after treatment with nintedanib, suggesting that the structural changes observed could also modulate the regenerative capacity of the muscles [20]. This potential anti-inflammatory effect of nintedanib has also been described in animal models of lung fibrosis and systemic sclerosis [42,63]. However, our data suggest that nintedanib cannot completely prevent macrophages infiltration in the muscle as we did not observe a significant reduction in the number of cells infiltrating the muscle in treated mice.

The assessment of the in vivo effect of drugs requires mouse models that resemble human disease as closely as possible. The murine model of Sgca^-/-^ displays a severe phenotype with clear muscle weakness already at 8 weeks that progressed over time [46,47]. At baseline, when the animals were 6 weeks of age, we did not observe significant differences in the muscle function tests between Wt and Sgca^-/-^ mice. However, at 11 weeks of age, we already observed a decline in muscle function, which was more evident at 16 weeks of age. Nintedanib improved functional muscle capacity assessed both by the treadmill and grip strength tests. However, nintedanib was not able to completely revert the pathogenic phenotype, suggesting that the degenerative process of the muscle is still active. These results raise the question of whether treating patients with an antifibrotic treatment is sufficient to completely change the disease’s natural history or whether the treatment just delays the progression of the disease. In our opinion, antifibrotic therapy could probably be considered as combined therapy with the new gene-delivery therapies or even as a treatment prior to gene therapy to condition muscle tissue for gene delivery. Recent results disclosed by companies running gene therapy clinical trials with DMD and sarcoglycan patients show that not all fibers express the transgene after treatment both in preclinical animal models and in patients. These findings suggest that various muscle fibers will continue to deteriorate, activating satellite cells that will proliferate and lead to loss of the transgene. Eventually, by the fusion of these satellite cells with the damaged myofibers, the expression of the transgene in the muscle is diluted. Therefore, it is possible that the fibrotic process will remain active supporting the use of combined therapeutic strategies using gene therapy and antifibrotic drugs. In agreement with this hypothesis, the combination of gene therapy using recombinant adeno-associated virus (rAAV) with antifibrotic or anti-inflammatory drugs, such as myostatin inhibitors, drugs modulating the neuronal nitric oxide synthase, microRNA-29 or VEGF expression, have shown better results than rAAV gene therapy alone [26,64,65,66]. This combined treatment seems to be more effective if administered early, probably because there is still a relatively preserved muscle environment resulting in better efficacy of the treatment [66].

In conclusion, treatment with nintedanib results in a functional muscle improvement and an attenuated muscle fibrosis and inflammation in Sgca^-/-^ mice. These results suggest that nintedanib could be considered as a potential therapy for patients with muscular dystrophies to be tested in clinical trials.

## Data Availability

The data that support the findings of this study are available from the corresponding authors, upon reasonable request.

## Supplementary Materials

The following supporting information can be downloaded at: https://www.mdpi.com/article/10.3390/biomedicines10102629/s1: Table S1. List of the 40 Cytokines, chemokines and acute phase proteins analyzed.; Table S2. List of the 84 fibrosis-related genes and 12 endogenous gene candidates analyzed.; Figure S1. Schematic representation of the *Ct* value of *Tbp* gene expression.; Figure S2. Schematic representation of weight gain after 10 weeks of treatment.; Figure S3. Cardiac performance analysis in Sgca^-/-^ mice.
