# Supplementary material for: Nintedanib Reduces Muscle Fibrosis and Improves Muscle Function of the Alpha-Sarcoglycan-Deficient Mice
Source: Biomedicines. 2022 Oct 19;10(10):2629. doi: 10.3390/biomedicines10102629 (PMC9599168; doi:10.3390/biomedicines10102629)
Supplement: Supplementary file 1 [file biomedicines-10-02629-s001.zip › Supplemental Tables.pdf]

Supplemental Table S1. List of the 40 Cytokines, chemokines and acute phase proteins analyzed.

Name			
CXCL131	IL-1ra	IL-16	CXCL9
C5a	IL-2	IL-17	CCL3
G-CSF	IL-3	IL-23	CCL4
GM-CSF	IL-4	IL-27	CXCL2
CCL1	IL-5	CXCL10	CCL5
CCL11	IL-6	CXCL11	CXCL12
ICAM-1	IL-7	CXCL1	CCL17
IFN-gamma	IL-10	M-CSF	TIMP-1
IL-1 alpha	IL-12	CCL2	TNF-alpha
IL-1 beta	IL-13	CCL12	TREM-1

Supplemental Table S2. List of the 84 fibrosis-related genes and 12 endogenous gene candidates analyzed. Endogenous control genes are in bold.

Name	Acession Number	Name	Acession Number	Name	Acession Number
Acta2	Mm00725412_s1	Itga1	Mm01306375_m1	Smad7	Mm00484742_m1
Agt	Mm00599662_m1	Itga2	Mm00434371_m1	Snai1	Mm00441533_g1
Akt1	Mm01331626_m1	Itga3	Mm00442910_m1	Sp1	Mm00489039_m1
Bcl2	Mm00477631_m1	Itgav	Mm00434486_m1	Stat1	Mm00439531_m1
Bmp7	Mm00432102_m1	Itgb1	Mm01253230_m1	Stat6	Mm01160477_m1
Cav1	Mm00483057_m1	Itgb3	Mm00443980_m1	Tgfb1	Mm01178820_m1
Ccl11	Mm00441238_m1	Itgb5	Mm00439825_m1	Tgfb2	Mm00436955_m1
Ccl12	Mm01617100_m1	Itgb6	Mm01269869_m1	Tgfb3	Mm00436960_m1
Ccl3	Mm00441259_g1	Itgb8	Mm00623991_m1	Tgfbr1	Mm00436964_m1
Ccr2	Mm00438270_m1	Jun	Mm00495062_s1	Tgfbr2	Mm00436977_m1
Col3a1	Mm01254476_m1	Lox	Mm00495386_m1	Tgif1	Mm01227699_m1
Cebpb	Mm00843434_s1	Ltbp1	Mm00498255_m1	Thbs1	Mm00449032_g1
Col1a2	Mm00483888_m1	Mmp13	Mm00439491_m1	Thbs2	Mm01279240_m1
Col3a1	Mm01254476_m1	Mmp14	Mm00485054_m1	Timp1	Mm00441818_m1
Ctgf	Mm01192932_g1	Mmp1a	Mm00473485_m1	Timp2	Mm00441825_m1
Cxcr4	Mm01996749_s1	Mmp2	Mm00439498_m1	Timp3	Mm00441826_m1
Dcn	Mm00514535_m1	Mmp3	Mm00440295_m1	Timp4	Mm01184417_m1
Edn1	Mm00438656_m1	Mmp8	Mm00439509_m1	Tnf	Mm00443258_m1
Egf	Mm00438696_m1	Mmp9	Mm00442991_m1	Vegfa	Mm01281449_m1
Eng	Mm00468256_m1	Myc	Mm00487804_m1	**18s rRNA**	Hs99999901_s1
Fasl	Mm00438864_m1	Nfkb1	Mm00476361_m1	**Gapdh**	Mm99999915_g1
Grem1	Mm00488615_s1	Pdgfa	Mm01205760_m1	**Hprt**	Mm00446968_m1
Hgf	Mm01135193_m1	Pdgfb	Mm00440677_m1	**Gusb**	Mm00446953_m1
Ifng	Mm01168134_m1	Plat	Mm00476931_m1	**Actb**	Mm00607939_s1
IL10	Mm00439614_m1	Plau	Mm01274460_g1	**B2m**	Mm00437762_m1
IL13	Mm00434204_m1	Plg	Mm00447087_m1	**Hmbs**	Mm00660262_g1
IL13ra	Mm00515166_m1	Serpina1a	Mm02748447_g1	**Ipo8**	Mm01255158_m1
IL1a	Mm00439620_m1	Serpine1	Mm00435860_m1	**Pgk1**	Mm00435617_m1
IL1b	Mm00434228_m1	Serpinh1	Mm00438058_g1	**Rplp2**	Mm00782638_s1
IL4	Mm00445259_m1	Smad2	Mm00487530_m1	**Tbp**	Mm00446973_m1
IL5	Mm00439646_m1	Smad3	Mm01170760_m1	**Tfrc**	Mm00441941_m1
ILk	Mm01274281_g1	Smad4	Mm03023996_m1		
Inhbe	Mm00434340_g1	Smad6	Mm00484738_m1		
