# Supplementary figures and images for: Nintedanib Reduces Muscle Fibrosis and Improves Muscle Function of the Alpha-Sarcoglycan-Deficient Mice
Source: Biomedicines. 2022 Oct 19;10(10):2629. doi: 10.3390/biomedicines10102629 (PMC9599168; doi:10.3390/biomedicines10102629)

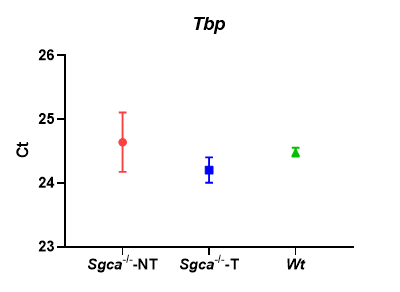

Supplement: Supplementary file 1 [file biomedicines-10-02629-s001.zip › Supplemental Figure S1.tif]

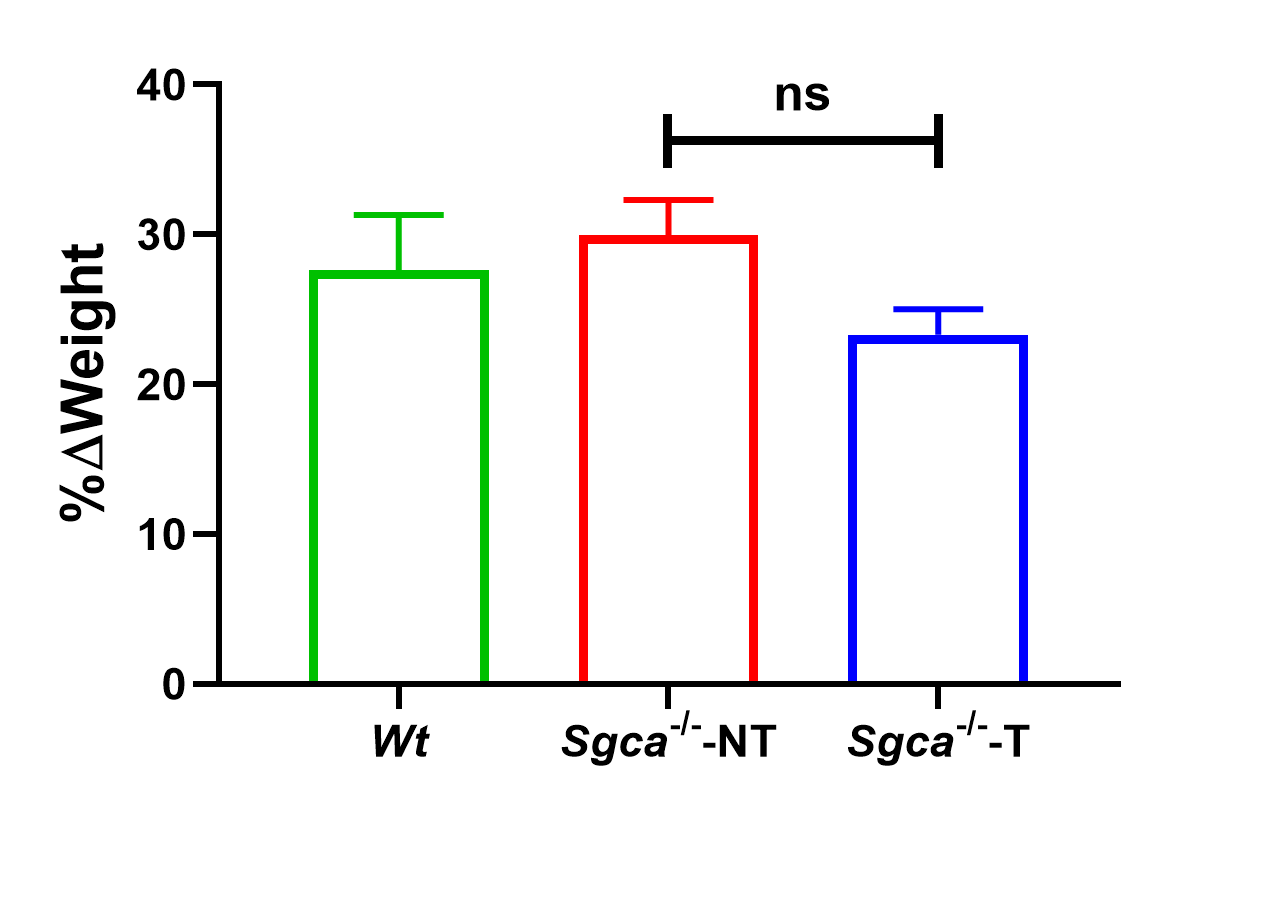

Supplement: Supplementary file 1 [file biomedicines-10-02629-s001.zip › Supplemental Figure S2.tif]

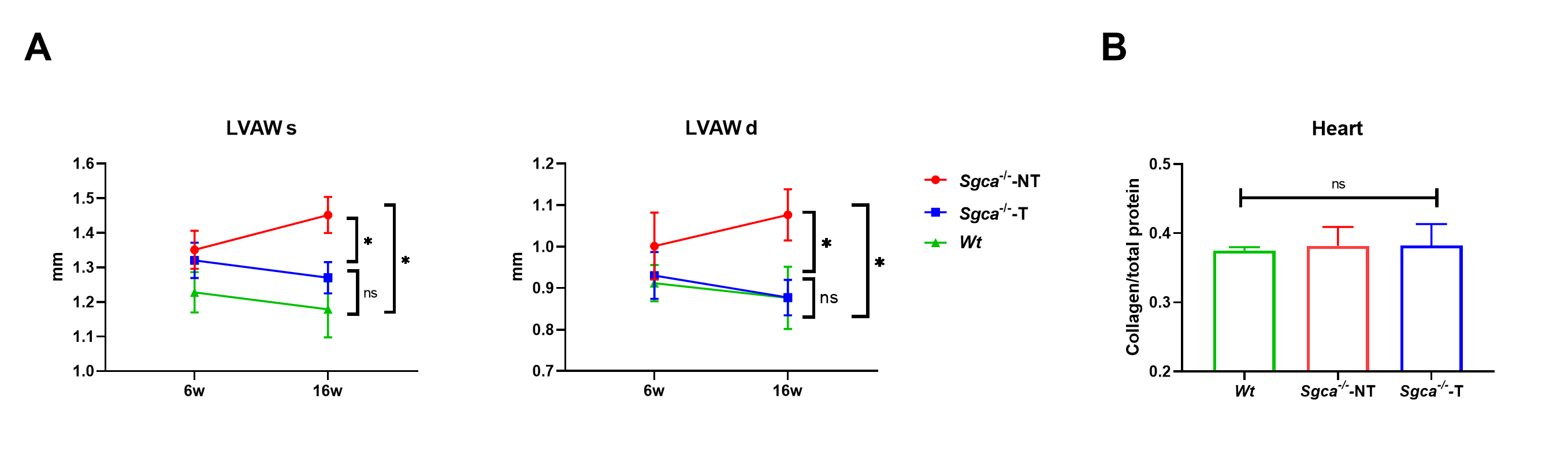

Supplement: Supplementary file 1 [file biomedicines-10-02629-s001.zip › Supplemental Figure S3.tif]
